# Development of pH-Sensitive Multiparticulates for Orally Disintegrating Tablets of Proton Pump Inhibitors: Physicochemical Characterization and Drug Release Studies

**DOI:** 10.3390/pharmaceutics17091187

**Published:** 2025-09-12

**Authors:** Mahendra Singh, Punna Reddy Ullapu, Arokia Vijaya Anand Mariadoss, Satyender Kumar, Sung Gu Kang

**Affiliations:** 1 Department of Biotechnology, Institute of Biotechnology, School of Life and Applied Sciences, Yeungnam University, Gyeongsan 38541, Republic of Korea; m.singh2685@gmail.com; 2 Research Institute, Dong-A ST Co., Ltd., Yongin 17073, Republic of Korea; punna3@gmail.com; 3 Orthopaedic Research Center, Kaohsiung Medical University, Kaohsiung 80708, Taiwan; mavijaibt@gmail.com; 4 Department of Biological and Chemical Sciences, School of Liberal Arts and Sciences, Mohanbabu University, Tirupati 517102, Andhra Pradesh, India; 5 School of Pharmacy, Sharda University, Knowledge Park III, Greater Noida 201310, Uttar Pradesh, India

**Keywords:** orally disintegrating tablet, dysphagia, enteric coating, direct compression, geriatric, pediatric, superdisintegrants

## Abstract

**Background/Objectives:** Enteric coating protects active pharmaceutical ingredients from gastric degradation, but conventional tablets may present swallowing difficulties in geriatric and pediatric patients. Hence, this study intended to develop pH-responsive multiparticulates, formulated into orally disintegrating tablets (ODTs), for targeted intestinal drug delivery in individuals with dysphagia. **Methods:** Multiparticulates were developed via sequential seal coating, drug layering, sub-coating, and enteric coating on inert cores using a fluidized bed coater (Pam Glatt, India; bottom spray). Selected enteric-coated batches were directly compressed into ODTs using microcrystalline cellulose (Avicel PH102) and mannitol (Pearlitol SD 160) as fillers, with Explotab^®^, Ac-Di-Sol^®^, or crospovidone M^®^ as superdisintegrants. **Results:** Multiparticulates exhibited mean diameters of 197.671–529.511 μm and span values of 0.603–0.838. Span value < 1, indicating a narrow size distribution. Electron microscopy confirmed the spherical morphology of Batches 7a and b. Enteric-coated batches (5b, 6, 7a, 7b) released ≤10% of the drug in 0.1 N HCl at 2 h. Optimized formulation ODT 7b released 7.904% of the drug under gastric conditions and 79.749% in phosphate buffer (pH 6.8) within 2.5 h, following first-order drug release kinetics. ODT 7b demonstrated hardness (2.538 ± 0.144 kg/cm^2^), wetting time (11.17 ± 1.051 s), friability (0.712%), and drug content (99.81 ± 1.01%) within acceptable limits. **Conclusions:** The pH-dependent multiparticulates provided sustained intestinal drug release and, when incorporated into ODTs, yielded a dosage form with a rapid wetting time and acceptable mechanical properties. This dosage form can offer a promising approach for improving compliance and therapeutic efficacy in patients with swallowing difficulties (dysphagia).

## 1. Introduction

Proton pump inhibitors (PPIs) are widely prescribed for the treatment of chronic gastritis, esophagitis, and gastroesophageal reflux disease (GERD) [1]. PPIs are also indicated for pathological hypersecretory conditions such as functional dyspepsia and Zollinger–Ellison syndrome, in which suppression of gastric acid secretion is essential [2,3]. In addition, PPIs are frequently employed as adjunctive therapy to prevent chronic type C gastritis associated with the prolonged use of nonsteroidal anti-inflammatory drugs (NSAIDs). PPIs are acid-unstable; therefore, for such types of drugs, an enteric coating is required to prevent the acidic degradation of the drug in the stomach and deliver it to a basic pH environment (the intestines, with a pH of 5.5 and above). Commonly used PPIs in clinical practice include omeprazole, esomeprazole, pantoprazole, and lansoprazole [4,5].

Furthermore, acid-related problems can be efficiently managed with pantoprazole. Pantoprazole is used as a monotherapy for treating esophagitis and maintaining gastroesophageal reflux disease (GORD) as part of a triple therapy for *Helicobacter pylori* eradication. It has been reported that pantoprazole has higher efficacy than histamine H2 antagonists and comparable efficacy to other PPIs [6]. In the treatment of moderate to severe GORD, oral pantoprazole 40 mg/day can be effective as other PPIs (omeprazole, omeprazole multiple unit pellet system, lansoprazole, and esomeprazole) [6]. Oral pantoprazole 20 or 40 mg/day is effective in healing and preventing NSAID-related ulcers. Pantoprazole inhibits gastric acid by blocking the H^+^/K^+^-adenosine triphosphatase enzyme system (the proton pump) of gastric parietal cells [7]. To obtain the highest efficacy of H^+^/K^+^-ATPases inhibition, PPIs should be administered 30–60 min before breakfast or before other substantial meals [8]. Pantoprazole sodium is a weakly basic compound, stable only in an alkaline environment. In addition, high acidity in the stomach poses a great challenge to the development of drug dosage forms for oral delivery of such a type of drug [7]. Hence, we need to develop a dosage form that protects the drug from degradation by stomach acid. Moreover, traditional monolithic tablets or capsules contain the drug in a single unit, hence the possibility of dose dumping. In contrast, multiparticulates consist of numerous small, discrete units, such as pellets, granules, or beads, loaded with the drug. The techniques used in multiparticulates systems include pellet formation, microparticle preparation, granule production, and nanoparticle formulation [9].

Pellets are small spherical particles ranging from 0.5 to 2.0 mm in diameter and are typically filled into capsules or compressed into tablets [10]. Enteric-coated pellets represent a sophisticated class of pH-dependent drug delivery systems offering various benefits over conventional formulations. Their primary advantage is site-specific drug release, hence ensuring the drug is released precisely where it is needed within the gastrointestinal tract (GIT) [11]. Additionally, these systems can improve stability and protect the drug(s) from degradation in the stomach’s acidic environment and are widely utilized for oral drug delivery, colon-targeted therapy, delayed release, and controlled-release formulations [12,13].

Eudragit^®^ derivatives, methacrylic acid copolymers such as methacrylic acid–methyl methacrylate copolymer (1:1) or Eudragit^®^ L100, and methacrylic acid–methyl methacrylate copolymer (1:2) or Eudragit^®^ S100 have commonly been used as pH-dependent coating agents to protect the drug core from the severe gastric environment [14] or as a hydrogel for medical devices [15]; Eudragit L30 D-55 is an anionic resin latex composed of methacrylic acid and ethyl acrylate in a 1:1 molar ratio and is used as an enteric coating agent for pH-sensitive formulations. The free carboxyl groups in Eudragit L30 D-55 are ionized above pH 5.5, and this the point at which the coating film begins to dissolve. In an acidic environment, the enteric coating film does not dissolve; hence, it protects the drug from acid degradation [16,17]. Thus, it is used to develop enteric-coated tablets, such as pellets of PPIs such as pantoprazole, to prevent acidic degradation in the GIT.

Moreover, various methods can be utilized to manufacture the pellets, such as direct pelletizing, suspension layering, powder layering, pelletization by expulsion and spheronization, pressure/balling, circular agglomeration, cryopelletization, liquefy spheronization, globulation or drop arrangement, and fluid bed coating. Out of them, fluidized bed coating is an effective technique for formulating pellets/microcapsules because of its adaptability, suitability in industrial-scale production, and simple process [18,19]. The preparation of microcapsules/pellets involves the use of drug crystals or neutral microparticles as the core material and polymers as the coating material in a fluidized bed coater [20,21]. Fluidized bed coaters are divided into three types according to their spraying method: top spray, bottom spray, and rotating tangent spray [19]. The latter two methods are commonly used to prepare pellets. The Wurster unit is a bottom spray type frequently employed for coating and granulation.

Additionally, dysphagia is the main problem for geriatric and pediatric patients in taking oral medication. It is defined as difficulty in swallowing [22]. Newman et al. [23] report that 15–40% of the population has signs of dysphagia. This number can likely be higher among older adults who suffer from diseases or chronic illnesses that contribute to dysphagia [24]. The presence of dysphagia often complicates health issues as well as the quality of life [25]. Also, dysphagia affects approximately 80% of individuals suffering from Parkinson’s disease, which comprises impaired movement of swallowing structures (e.g., lingual movement), timing abnormalities of swallowing events (e.g., prolonged oral transit), oropharyngeal residue, and airway invasion [26,27,28,29]. Hence, innovative formulations, for instance, orally disintegrating tablets (ODTs), need to be developed because traditional oral dosage forms, such as capsules and tablets, pose difficulties in swallowing for pediatric and geriatric populations.

Thus, pharmaceutical drug delivery scientists have designed orally disintegrating tablets (ODTs), a novel oral dosage form that dissolves quickly in saliva, typically within a few seconds to minutes, and eliminates the need for water to meet these medical needs. The fast-dissolving property of these dosage forms is attributed to the swift ingress of water into the tablet matrix, resulting in rapid disintegration in the mouth [30]. Methods that can be used to develop these formulations are freeze-drying or lyophilization, spray drying, sublimation, and direct compression [31].

In this study, enteric-coated multiparticulates were developed by employing different solution/suspension layering on a nonpareil core material in a bottom spray fluidized bed coater (Pam Glatt, India). In addition, to utilize the numerous benefits of unit dosage forms, such as cost effectiveness, ease of manufacturing, patient compliance, and various pharmaceutical industry applications, ODTs were formulated by employing directly compressible materials with different super-disintegrating agents. The enteric-coated pellets and ODT with pellets were evaluated for the various physicochemical and in vitro release studies. Hence, the study aimed to develop the innovative drug-containing pH-sensitive enteric-coated multiparticulates comprising ODT formulations that effectively protect the drug from the gastric acidic environment. This innovative product can benefit the dysphagia populations, regardless of the age of the population (pediatric or geriatric), in relieving flatulence problems or other gastric problems such as ulcers, gastritis, GORD, or esophagitis.

## 2. Materials and Methods

Crosslinked PVP and PVP K-29/32 (ISP Technologies Inc., Wayne, NJ, USA), ethyl cellulose (BDH Chemicals Ltd., Poole, Dorset, UK), HPMC-E5 (Huzhou Hopetop, Pharmaceutical Co., Huzhou, China), HPMC-E50LV, HPMC-E4M, HPMC-K100LV, and HPMC-K4M (Ranbaxy Laboratories Ltd., Gurgaon, India), spray-dried lactose (Ind-Swift Ltd., Parwanoo, India), Pearlitol SD 160 (Roquette, Lestrem, France), Eudragit L100 (Acrycoat), and Eudragit L30 D-55 (Corel Pharma Chem, Ahmedabad, India) were generously gifted. Sodium starch glycollate, croscarmellose sodium (Loba Chemie, Mumbai, India) and PEG-6000 (S.D. Fine chemicals Ltd., Mumbai, India) were purchased. All other ingredients and reagents were of analytical grade.

### 2.1. Preparation of Multiparticulates

The preparation of the multiparticulates is a four-step process. The procedure for coating is as follows: Step 1: Seal coat with ethyl cellulose onto nonpareil pellets (microcrystalline cellulose PH112); Step 2: Drug layering; Step 3: Sub-coat or protective layering with HPMC and; Step 4: Enteric coating with Eudragit L 100 (non-aqueous polymer) and Eudragit L30 D55 (aqueous suspension).

All the steps were completed in the fluidized bed mini Glatt (Pam Glatt, Pune, India) coater using the bottom spraying technique, with a Wurster insert and a nozzle size of 0.3 mm. The coating solution was applied by using a peristaltic pump with a silicon pipe that had a hose size of 2.0 mm (internal diameter), and the quantity of applied coating was measured using a balance (Mettler, Mumbai, India). The product temperature and inlet temperature were monitored through a temperature sensor fitted within the fluidized bed instrument.

#### 2.1.1. Seal Coating

Seal coating was performed with a 4% *w*/*v* solution of ethyl cellulose in isopropyl alcohol on nonpareil pellets (passed #60). The required quantity (100 g) of nonpareil core material was loaded into the bottom spray fluidized bed coater and processed as per the coating parameters presented in Table 1. The procedure was continued until the overall weight gain (2%) of nonpareil pellets was achieved. Subsequently, seal-coated pellets were dried in a fluidized bed coater at 40 °C for 15–20 min.

#### 2.1.2. Drug Layering

After passing #60, seal-coated pellets were placed into the fluidized bed coater, and drug layering was performed onto the seal-coated core material. The fresh drug solution (15% *w*/*v*) was prepared by dissolving the drug in distilled water containing binder (PVP K29/32) in a concentration of 0.5 to 1.0% *w*/*v*. A weight gain of 22.60% of total seal-coated pellets was achieved under controlled process parameters, as shown in Table 1. Finally, the drug-coated pellets were dried in a fluidized bed coater at 30 °C for 15–20 min and stored in an airtight light-resistant container for further use.

**Table 1:** Process parameters for developing multiparticulates.

Parameters	Process Steps				
	Seal Coat	Drug Layering	Sub-Coat	Enteric Coat (Non-Aqueous)	Enteric Coat (Aqueous)
Inlet temperature (°C)	70	70	70	70	70
Product temperature (°C)	26–28	30–32	30–32	28–30	30–32
Pump speed (RPM)	0.5–1.1	0.4–0.95	0.5–1.15	0.40–0.90	0.50–0.90
Spray rate (g/min)	2–5	2–4	2–6	2–4	2–4
Fluidization rate (MPa)	0.4–0.60	0.50–0.7	0.40–0.60	0.40–0.60	0.40–0.60
Atomization rate (MPa)	0.60–1.0	0.60–1.10	0.70–1.20	0.60–0.95	0.50–1.10
Nozzle diameter (mm)	0.3	0.3	0.3	0.3	0.3
Inlet air volume (m^3^/h) at 6 bars	60–64	60–64	60–64	60–64	60–64

#### 2.1.3. Sub-Coating

Using sub-coating polymers, as indicated in Table 2, a protective coating was applied to drug-layered pellets (passed #40 to remove any agglomerates) in the third step. Based on the viscosity of the polymers, a sub-coating solution was made in distilled water at a concentration of 0.5–5% *w*/*v*. Before coating, the sub-coating solution was filtered through a muslin cloth to remove any lumps. It was prepared two to three hours before the coating process. The drug-coated pellets were placed into a fluidized bed coater and coated using the parameters as listed in Table 1. A weight gain of 2–15% *w*/*w* of drug-coated pellets was achieved, as seen in Table 2. After processing, sub-coated pellets were dried in a fluidized bed coater at 40 °C for 15–20 min and stored in an airtight light-resistant container for further use.

#### 2.1.4. Enteric Coating

The next step was an enteric coating on the sub-coated pellets. Sub-coated pellets were screened through a #40 mesh to remove any lumps, then loaded into a fluidized bed coater, and processed according to the parameters listed in Table 1. Enteric-coated multiparticulates were prepared by applying Eudragit L100 (non-aqueous coating) and Eudragit L-30 D55 (30% *w*/*v*, aqueous coating) to achieve a weight gain of 10.0–35.0% *w*/*w* (Table 2). For the non-aqueous coating, dibutyl phthalate was used as the plasticizer, and for the aqueous coating, PEG 6000 was added as a plasticizer at a concentration of 10% *w*/*w* for each polymer based on dry polymer weight. The non-aqueous coating solution was prepared using a solvent mixture of isopropyl alcohol and acetone in a 1:1 volume ratio under continuous stirring. Ready-made Eudragit L-30 D55 was diluted with distilled water. The solutions were then filtered through muslin cloth to remove any lumps. The 6.0–10.0% *w*/*v* concentration of enteric coating solution was used for coating purposes. The solution was prepared 2–3 h before coating. The process (Table 1) was performed to achieve the required weight gain of the enteric coat (Table 2). Further, enteric-coated multiparticulates were dried in a fluidized bed at 40 °C for 15–20 min. After that, the multiparticulates were subjected to curing in the oven at 40–45 °C for 3–4 h to complete film formation and drying. Finally, they were stored in a vacuum desiccator in an airtight, light-resistant container until the next use.

### 2.2. Characterization of Enteric-Coated Multiparticulates

#### 2.2.1. Size Analysis

The size analysis of all batches of the multiparticulate pellets was performed using a Malvern Mastersizer 2000 (Malvern, Worcestershire, Grovewood Road, UK), employing the dry dispersion technique at an air pressure of 0.22–0.40 bar and a feed rate of 29–42%. The mean diameter [D (0.5)] and span values were evaluated. The size distribution graphs were automatically generated.

#### 2.2.2. Morphology and Shape Analysis

A Scanning Electron Microscope (SEM) was used to check the morphology of the optimized batch of pellets (JSM 6100, Tokyo, Japan). The pellets were coated with a thin gold/palladium layer and then examined at 5 kV at various magnification levels from 130× to 270×.

Following this, the shape analysis was performed by using an Olympus CH-20i-Tr optical microscope (Olympus, Mumbai, India) with a projection system at 10× magnification. The diameters of 20 pellets were measured randomly from each batch and noted to calculate different shape parameters (elongation, rectang, and roundness), employing the following formulae:

$$Elongation=\frac{Maximum\;radius}{Minimum\;radius}$$

$$Rectang=\frac{Area}{4\times Maximum\;radius\times Minimum\;radius}$$

$$Roundness=\frac{Area}{\pi \times {Maximum\;radius}^{2}}$$

#### 2.2.3. Porosity and Density

Porosity and densities of the multiparticulates were determined as per the reported method [32]. The bulk density of the multiparticulates was analyzed by gently pouring 25 g of pellets into a graduated measuring cylinder. The volume thus occupied was noted (bulk volume) to calculate the bulk density. Further, the cylinder was then tapped 500 times from a height of 14 mm, and the volume was noted. It was tapped an additional 750 times or until no further decrease in volume was observed, and the final volume (tapped volume) was used to calculate the tapped density. All the determinations were performed in triplicate.

Bulk density, tapped density, and porosity were calculated by applying the following formulas:

$$Bulk\;density(\delta )=\frac{M}{Vb}$$

$$Tapped\;density(\delta )=\frac{M}{Vp}$$

$$\%\;Poroity\;(\varepsilon )\;=\frac{Vb-Vp}{Vb}\times 100$$

*Vb* = Bulk volume of the multiparticulates;

*Vp* = True/Tap volume of the multiparticulates;

*M* = Mass of multiparticulates.

#### 2.2.4. Hausner Ratio and Carr’s Index

Both parameters indicate the flow properties of a powder or granular material. Hausner ratio and Carr’s index were calculated using the following formulas [33]:

$$Hausner\;ratio=\frac{\rho tapped}{\rho bulk}$$

$$Carr\prime s\;index=\left[1-\frac{\rho bulk}{\rho tapped}\right]*100$$

where ‘*ρ*bulk’ and ‘*ρ*tapped’ are the bulk and tapped densities, respectively.

#### 2.2.5. Angle of Repose and Flow Rate

The flow properties were measured in terms of angle of repose and flow rate. For the determination of the angle of repose, 25 g of pellets was allowed to flow freely through a funnel onto a smooth, vibration-free, and horizontal surface from a height of 3 cm. The diameter and height of the heap were measured, and the angle of repose was calculated using the following formula:

$$\theta ={tan}^{-1}\frac{h}{r}$$

where ‘*θ*’ is the angle of repose, and ‘h’ and ‘r’ are the height and the radius of the heap, respectively.

The flow rate was calculated as the time taken for 10 g of pellets to flow through a funnel. The determinations for the angle of repose and flow rate were performed in triplicate, and the results are presented as the mean with standard deviation of each batch.

#### 2.2.6. Differential Scanning Calorimetry (DSC)

DSC analysis was carried out Thermal Analysis Q20 differential scanning calorimeter to examine the physical state of the drug in the pellet formulations. The pure drug and pellets of the optimized batch were placed in separate aluminum pans and sealed. Then they were placed and heated at 5 °C/min in the range of 10–300 °C, using an empty sealed pan as a reference and dry nitrogen as the purge gas.

#### 2.2.7. Drug Content

An accurately weighed amount of the multiparticulates of each batch equivalent to 22.6 mg of drug was dispersed in 100 mL of phosphate buffer (pH 6.8) in a 100 mL volumetric flask. Further, they were sonicated in a bath sonicator until the multiparticulates completely dissolved. Afterwards, the aliquot sample was centrifuged for 10 min at 4000 rpm. The supernatant was collected, filtered, subsequently diluted, and the drug content was determined spectrophotometrically at a wavelength of 290 nm (UV-vis spectrophotometer, Genesys, Waltham, MA, USA). The drug content of each batch was determined in triplicate.

#### 2.2.8. In Vitro Drug Release from Multiparticulates

The in vitro release profile of the multiparticulates pellets was determined in hexaplicate using a USP type II apparatus containing 750 mL of 0.1 N HCl maintained at 37 ± 0.5 °C for 2 h and then further in phosphate buffer pH 6.8 (with 250 mL addition of 0.2 M trisodium phosphate in 750 mL of 0.1 N HCl). The stirring speed was kept at 100 rpm. Samples were withdrawn at various time intervals over 12 h and replaced with the same release medium. The samples were analyzed by using a UV spectrophotometer at 290 nm to calculate the drug release. The graph was plotted as mean and standard deviation.

#### 2.2.9. Drug Release Kinetics of Multiparticulates

To study the mechanism of drug release from the prepared multiparticulates, the release kinetics data were evaluated by using zero-order (Equation (1)), first-order (Equation (2)), Higuchi’s square root of time equation (Equation (3)), Korsemeyer and Peppas Equation (Equation (4)), and Hixson–Crowell’s cube root of time equation (Equation (5)) [34]. The goodness of fit was assessed by comparing the correlation coefficient (r^2^) values for respective batches.

M_t_ = M_0_ + k_0_t (1)

where M_t_ is the amount of drug dissolved in time t, M_0_ is the initial amount of drug in the solution, K_0_ is the zero-order release rate constant, and t is the release time.

logM_t_ = logM_0_ − k_t_t (2)

where M_t_ is the amount of drug dissolved in time t, M_0_ is the initial amount of drug in the solution, K_t_ is the first-order release rate constant, and t is the release time.

M_t_ = k_h_√t (3)

where M_t_ is the amount of drug dissolved in time t, k_h_ is the Higuchi dissolution constant, and t is the release time.

M_t_/M_∞_ = kt^n^ (4)

Here, M_t_ and M_∞_ are the absolute cumulative amount of drug released at time t and infinite time, respectively; k is a constant which incorporates structural and geometric characteristics of the formulation, and n is the drug release exponent (DRE), revealing the mechanism of drug release. The values of DRE assigned to a cylinder are 0.45 for Fickian diffusion and 0.45 < n < 0.89 for non-Fickian diffusion, respectively, while for the sphere, it is 0.43 for Fickian diffusion and 0.43 < n < 0.85 for non-Fickian diffusion, respectively.

Hixson–Crowell’s cube root of time equation is as follows:

M_0_^1/3^ − M_t_^1/3^ = k_s_t (5)

where M_0_ is the initial amount of drug in the formulation, M_t_ is the amount remaining at any time t, and k_s_ is the constant incorporating the surface-volume relation.

### 2.3. Preparation of Orally Disintegrating Tablets (ODTs)

The compositions of ODTs are presented in Table 3. ODTs were developed for those enteric-coated batches that showed near or <10% drug release in acid media (0.1 N HCl, pH 1.2) at 2 h. By using three different superdisintegrants (Table 3), the ODT 5a, ODT 5b, ODT 6, ODT 7a, and ODT 7b were formulated with optimized enteric-coated pellet batches, i.e., Batch 5a, Batch 5b, Batch 6, Batch 7a, and Batch 7b, by the direct compression method.

Briefly, mannitol DC (Pearlitol SD 160, MTL DC), microcrystalline cellulose PH102 (MCC PH102, Avicel PH102), and the multiparticulates of each batch were previously passed through #40. The quantities of ingredients were weighed as shown in Table 3 and mixed in a polybag separately with each enteric-coated batch for 10–15 min. Further, the disintegrating agents, i.e., sodium starch glycollate (SSG or Explotab^®,^, Loba Chemie, Mumbai, India), croscarmellose sodium (CCS or Ac-Di-Sol^®^, Loba Chemie, Mumbai, India), crosslinked polyvinylpyrrolidone (CL-PVPor Crosspovidon M^®^, Loba Chemie, Mumbai, India) passed through #60, sweetener and citric acid (previously pulverized in pestle mortar, #80), and flavor (#60) were weighed and mixed in a polybag for 10–15 min orderly. The powder mix was lubricated by adding magnesium stearate and then mixed for 2–3 min. Three ODT formulations with each pellet batch were prepared to evaluate the different disintegrant effects (Table 3). The tablets were compressed in a 16-station automated tablet press machine (Modern Engineering, New Delhi, India) by using a 13.0 mm round-shaped flat punch with a score on the upper punch. The average weight of the tablets was kept at 630.0 mg.

### 2.4. Evaluation of ODTs

#### 2.4.1. Weight Variation

Twenty tablets were selected randomly from each formulation and weighed individually using a weighing balance (Mettler, Mumbai, India). The individual weights were compared with the average weight to calculate the variation in the weight of tablets.

#### 2.4.2. Diameter and Thickness

All the tablet batches were evaluated for diameter and thickness by using Vernier calipers. The diameter and thickness of the six tablets from each batch were measured, and the average value reported.

#### 2.4.3. Hardness and Friability

The hardness of the six tablets from each batch was measured using the Monsanto Hardness Tester (Campbell Electronics, Mumbai, India).

The friability of the tablets was measured using a USP-type Roche friabilator in triplicate (Tropical Lab Equipment, Mumbai, Maharashtra, India). Pre-weighed tablets (≥6.5 g) were placed in the plastic drum of the friabilator instrument. A 100 revolution was rotated at a speed of 25 rpm for 4 min. The tablets were then de-dusted, reweighed, and the percentage weight loss (friability) was calculated as follows:

$$\%Friability=\frac{Initial\;weight-final\;weight}{Initial\;weight}*100$$

#### 2.4.4. Drug Content Uniformity

For the content uniformity test, 20 tablets were weighed and pulverized to a fine powder. A quantity of powder equivalent to 22.6 mg of drug was extracted into phosphate buffer (pH 6.8) and centrifuged for 10 min at 4000 rpm. The supernatant was collected and subsequently diluted. The drug content was determined by measuring the absorbance at 290 nm after appropriate dilution with the same medium. The drug content was determined using a regression equation. The mean percent drug content was calculated as an average of three determinations.

#### 2.4.5. Wetting Time

A conventional method was utilized to measure the wetting time of the ODT as reported previously [35]. A piece of tissue paper folded twice was placed in a small culture Petri dish of diameter 6.5 cm that contained 6.0 mL of water. The wetting time was determined by placing the tablet into a prepared Petri dish and observing the time to complete wetting of the tablet. This experiment was repeated in triplicate.

#### 2.4.6. In Vitro Drug Release from ODT

The in vitro release profile of the tablets was determined by using a USP type II apparatus at 37 ± 0.5 °C and 100 rpm in 750 mL of 0.1 N HCl for 2 h, then in phosphate buffer pH 6.8 (with the addition of 250 mL of 0.2 M tri-sodium phosphate in 750 mL 0.1 N HCl). Samples were withdrawn at various time intervals over 12 h and replaced with the same thermoregulated release medium. The samples were analyzed at 290 nm using a UV-VIS spectrophotometer, and the drug release was calculated. The release study was performed in hexaplicate. The graph was plotted as mean and standard deviation.

#### 2.4.7. Drug Release Kinetics of ODT

The drug release kinetics of ODTs were determined similarly as mentioned in Section 2.2.9. Drug Release Kinetics of Multiparticulates.

## 3. Results

### 3.1. Multiparticulates Size Distribution Analysis

The size distribution of the multiparticulates should be as narrow as possible, as it ensures a minimum variation in coating thickness and coating performance within the batch. If the multiparticulates are intended for compression, a wide size distribution may lead to segregation and variations in content uniformity. Mean particle size and span values for prepared batches of multiparticulates are depicted in Table 4.

**Figure 1:**
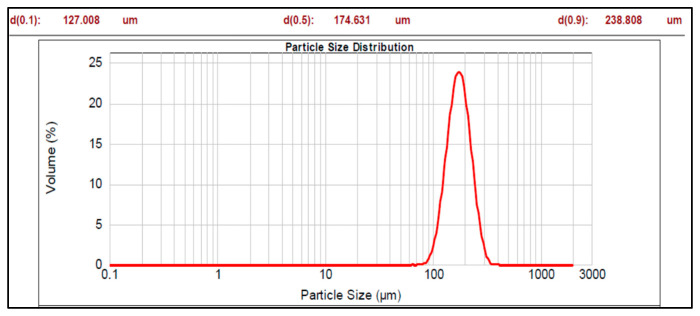
Particle size distribution of core material.

**Figure 2:**
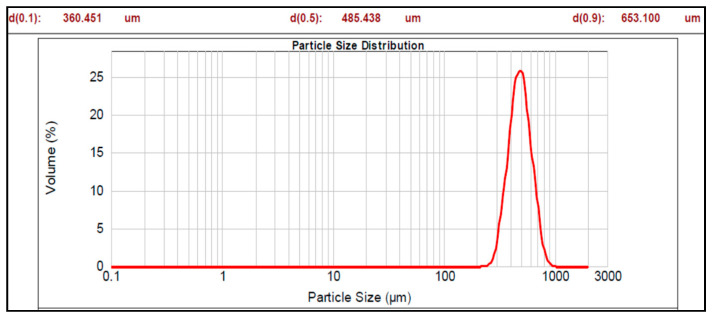
Particle size distribution of pellet Batch 7a.

**Figure 3:**
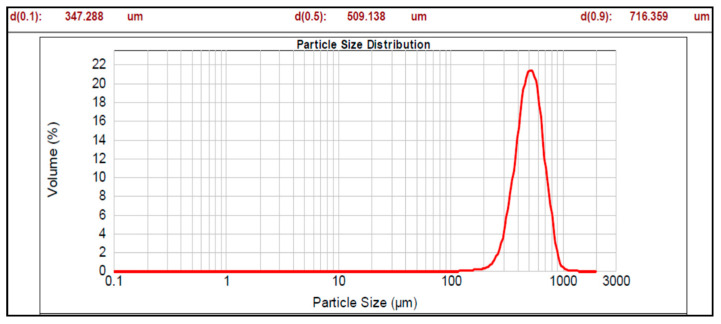
Particle size distribution of pellet Batch 7b.

The graphs of particle size distribution of the drug, core material (nonpareil), and optimized batches are shown in Figure 1, Figure 2 and Figure 3.

The mean diameter of all the prepared pellet batches (Batch 1 to 7b) of multiparticulates varied from 197.671 to 529.511 µm with span values of 0.603 to 0.838 (Table 4). Among the prepared batches, Batch 2 represented a high span value, i.e., 0.838, and Batch 7a showed the lowest span value (0.603). Higher span values are representative of non-uniform size distribution, while smaller span values are results of uniformity in size distribution. A high mean particle size distribution (529.511 µm and span value 0.763) was observed for Batch 5a, while Batch 5b showed nearly the same particle size (525.395 µm) as Batch 5a but a lower span value of 0.653 compared to Batch 5a. Batch 5b showed indicates a narrower size distribution than Batch 5a. It may be due to static charge development during the enteric coating step (non-aqueous coating), which may cause non-uniform coating and results in deviation in size distribution in Batch 5a. The optimized Batches 7a and 7b showed the particle size (485.438 and 509.139 µm, respectively) with span values of 0.603 and 0.625, respectively. Hence, it can be concluded that particle size varied with the coating weight of sub-coating and type of enteric coating polymers, as shown in Table 4.

### 3.2. Morphology and Shape Analysis of Multiparticulates Pellets

**Figure 4:**
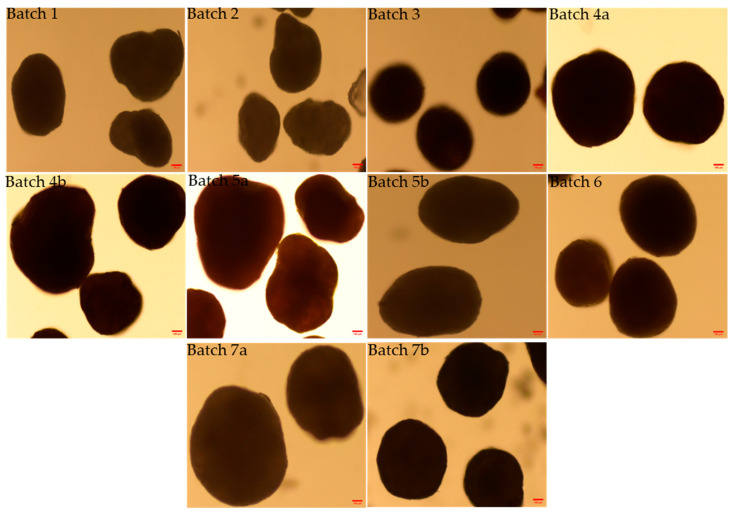
Optical microscopic images of various enteric-coated multiparticulates pellets (scale bar = 100 μm).

**Figure 5:**
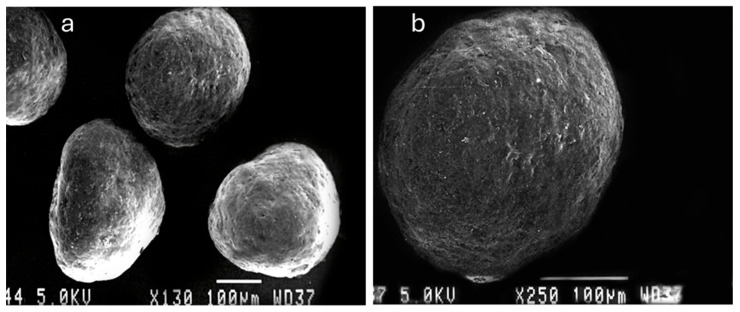
SEM images of Batch 7a (**a**) 130× and (**b**) 250×.

**Figure 6:**
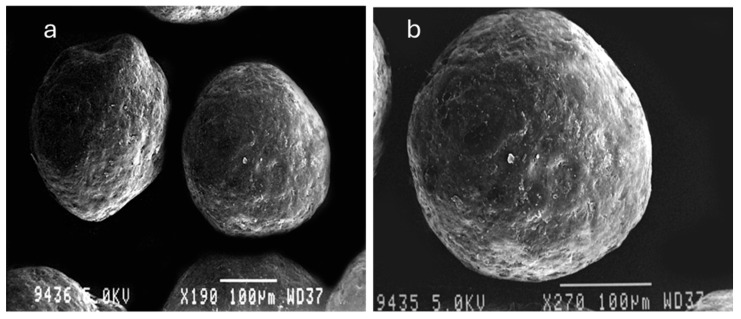
SEM images of Batch 7b (**a**) 190× and (**b**) 270×.

The morphology and shape analysis of the multiparticulates pellets was performed by optical microscopy images and scanning electron microscopy analysis (Figure 4, Figure 5 and Figure 6). The results of the various shape factors are listed in Table 5.

For all the prepared pellet batches, the roundness factor ranged from 0.781 to 0.917, indicating that all the pellets formed were almost circular (Table 5). Furthermore, the elongation factor ranged from 1.098 to 1.298, and the rectang observed between 0.766 and 0.833 for all the pellet batches. The highest roundness value (0.917) was obtained for Batch 7b, which contains 15% *w*/*w* low viscosity HPMC E5 as the sub-coating material and 35% *w*/*w* aqueous enteric coat. The lowest roundness value (0.781) was shown by Batch 4b, which was coated with 4% *w*/*w* HPMC E4M and 35% *w*/*w* non-aqueous enteric coating. Also, the SEM image of optimized Batch 7b showed the smooth surface coating and roundness of multiparticulates. Therefore, it can be concluded that the roundness increased with the progressive increase in percentage weight gain of the sub-coating and type of enteric coating (Table 2 and Table 5). This was also confirmed with microscopic images (Figure 4, Figure 5 and Figure 6).

### 3.3. Flow Properties Parameters

Bulk density indicates the extent of densification or compactness of a substance. The bulk densities of all batches varied from 0.677 to 0.798 g/cm^3^, tapped densities from 0.741 to 0.869 g/cm^3^, and porosity from 5.57 to 10.52% as shown in Table 6.

Among the prepared pellet batches, the lowest bulk density was obtained for Batch 3 (0.677 g/cm^3^), while the highest bulk density was observed for Batch 5b, i.e., 0.798 g/cm^3^. The optimized batch 7b showed the bulk density and tapped density 0.741 and 0.792 g/cm^3^, respectively.

In addition, the Hausner ratio for all pellet batches ranges from 1.017 to 1.141, and Carr’s index for various batches ranges from 5.582 to 13.362 (Table 6). The Carr’s index is also called “percent compressibility”. Both Carr’s index and the Hausner ratio are based on the difference between bulk and tapped densities, and give information about flow properties. Lower values for both indicate less resistance to flow and indicate good or excellent flow properties. The standard range of the Hausner ratio and Carr’s index is shown in Table 7. According to the Hausner ratio, all the prepared multiparticulates batches showed excellent and good flowability. Similarly, the Carr’s index indicated excellent and good flow properties for the prepared multiparticulates batches.

### 3.4. Angle of Repose and Flow Rate

The results of the angle of repose and flow rate for all multiparticulate batches are listed in Table 8. Furthermore, the flow properties of developed multiparticulates batches were measured by calculating the angle of repose and flow rate. Low values of angle of repose (<30°) are an indication of excellent flow properties (Table 7) of prepared multiparticulates.

Even though all batches showed good flow properties, the best flow properties were found for optimized Batch 7b with an angle of repose of 18.29° and flow rate 4.16 g/s as shown in Table 8. Batches prepared from aqueous dispersion enteric coating, i.e., Batch 3, Batch 4a, Batch 5b, Batch 6, Batch 7a, and Batch 7b, exhibited a low angle of repose values, hence showing good flow properties. Non-aqueous-coated multiparticulate batches, i.e., Batch 1, Batch 2, Batch 4b, and Batch 5a, showed slightly higher angles of repose than aqueous-coated batches. This might be due to the development of static charge between the particles during the air suspension coating of non-aqueous multiparticulates batches. Therefore, it can be concluded that all batches that exhibited a low angle of repose and good flow properties were prepared from an aqueous dispersion of enteric coating polymer rather than batches prepared from non-aqueous enteric coating materials.

### 3.5. Drug Content Uniformity

The results of the drug content are presented in Table 8. The analysis of drug content was performed for all the prepared multiparticulates batches and found in the range of 97.83 ± 1.41 to 99.78 ± 1.28 for all the multiparticulates batches.

### 3.6. Differential Scanning Calorimetry (DSC)

The thermal analysis technique known as DSC unveils both physical and chemical alterations in a material by measuring the difference in heat flow between a sample and a reference as a function of temperature. DSC is commonly utilized in research, quality control, and product development in various fields, like polymers and pharmaceuticals. It can determine thermal properties, for instance, melting points, crystallization behavior, glass transitions, and chemical reactions by observing heat absorption or release [36].

**Figure 7:**
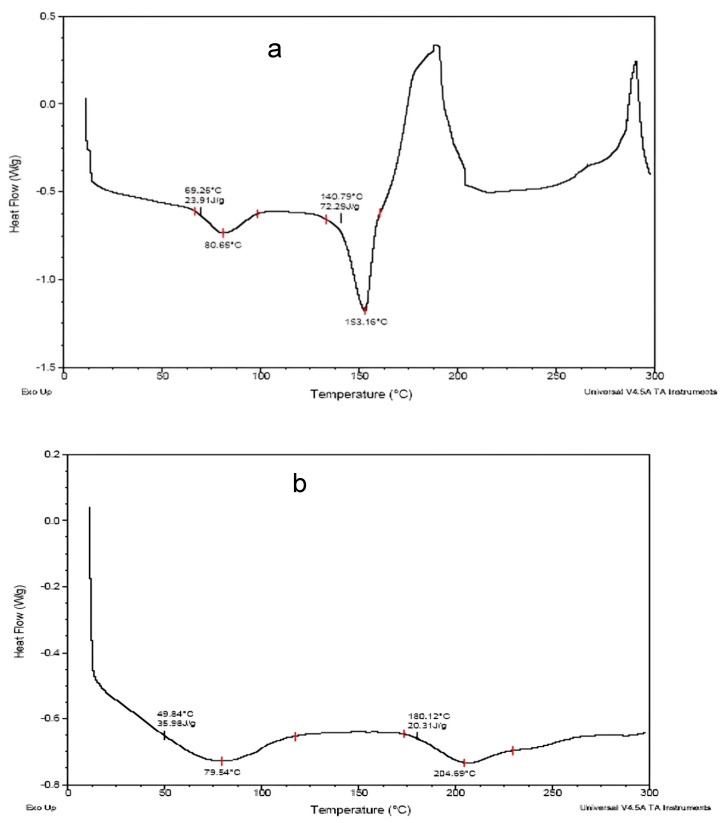
DSC thermogram of (**a**) pure drug and (**b**) a multiparticulates pellet of Batch 7b.

DSC thermogram peaks at 65.29 °C (enthalpy 23.31 J/g) and 80.65 °C, indicating glass transitions (Tg) or recrystallization events of the drug (Figure 7a). Peaks at temperatures of 140.79 °C (enthalpy 72.29 J/g) and 153.16 °C indicate strong endothermic peaks characteristic of a melting transition (Tm), most likely of a crystalline component. A peak at 153.16 °C corresponding to the melting point of the drug, as shown in Figure 7a.

DSC thermogram peaks at 49.84 °C (enthalpy 35.58 J/g), with potentially 79.54 °C representing cold crystallization transitions, while at 160.12 °C (enthalpy 26.31 J/g) and 204.59 °C broader peaks were observed and suggest the amorphous or semi-crystalline behavior of the drug (Figure 7b). The reduced enthalpy (J/g) and broadness indicate less crystallinity. Hence, the drug may be molecularly dispersed or complexed with polymers during the sub-coating process, consequently reducing the crystallinity and shifting the melting point.

### 3.7. In Vitro Dissolution Studies of Multiparticulates

**Figure 8:**
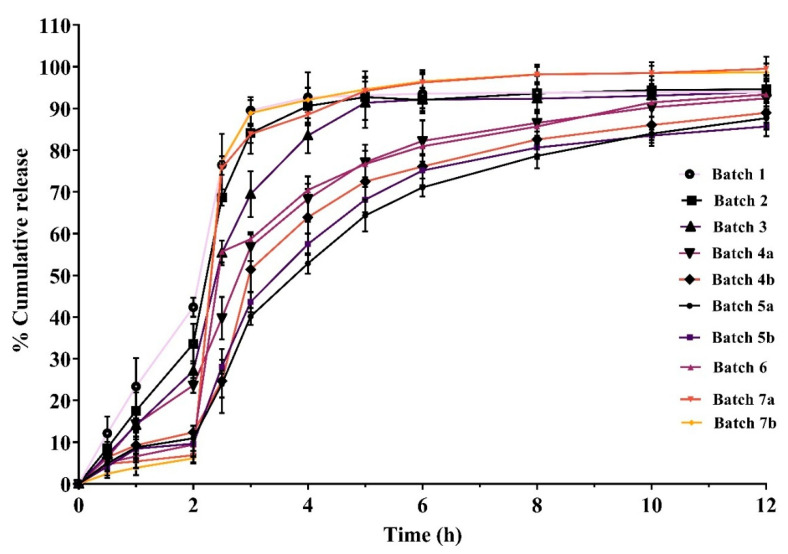
Cumulative percent drug release from different multiparticulates pellet batches.

To determine the impact of distinct excipients in varying ratios, coating compositions, and coat thicknesses on the drug’s release from the produced formulations, dissolution experiments are conducted. Hence, the type and coating level (%) of the enteric coating polymer as well as the multiparticulate pellets’ acid resistance when coated with aqueous and non-aqueous enteric coating polymers were identified. The dissolution profiles of enteric-coated pellets are depicted in Figure 8.

Batch 1, Batch 2, and Batch 3 exhibited drug release of 42.376%, 33.601%, and 27.085% in acidic medium (0.1 N HCl, pH 1.2) for 2 h, respectively (Figure 8). The high amount of drug release in acidic medium could be due to low percentages of sub-coating and enteric coating. A further change in the coating polymer concentration was performed to achieve a drug release of less than or equal to 10% in acidic medium at 2 h.

Hence, Batch 4a, Batch 4b, and Batch 5a were prepared, and in vitro drug release was shown to be lower than previous batches, i.e., 23.634%, 12.371%, and 10.941% in acidic medium for 2 h, respectively (Figure 8). Batch 4a displayed 23.634% drug release in acidic conditions at 2 h; this might be due to the incomplete/insufficient coating of the sub-coated particle. Batch 4b released 12.371% of the drug in acidic medium at 2 h, less than Batch 4a. This could be due to the increase in enteric coat level in Batch 4b (35% *w*/*w*). Furthermore, Batch 5a exhibited 10.941% drug release in acidic medium at 2 h, less than Batch 4b. This might be due to a higher sub-coating level, which covered the drug layer and retarded the drug release (Table 2 and Figure 8).

As the drug release in acidic medium was observed to be higher than the official compendia limit (<10%), further modification in coating level and polymer selection was performed in subsequent batches. Subsequently, the multiparticulates Batch 5b, Batch 6, Batch 7a, and Batch 7b were prepared, which showed drug release of 9.648%, 9.412%, 6.877%, and 6.136% in acidic medium at 2 h, respectively (Figure 8). Hence, these batches were used to develop the ODTs.

### 3.8. Kinetic Model for Drug Release from Various Multiparticulates Pellet Batches

The multiparticulates pellets Batch 1, Batch 2, Batch 3, and Batch 4a followed the Korsmeyer–Peppas release model, while Batch 4b, Batch 5a, Batch 5b, Batch 6, Batch 7a, and Batch 7b observed first-order release kinetics. Batch 4b, Batch 5a, Batch 5b, Batch 6, Batch 7a, and Batch 7b confirmed the concentration-dependent drug release (Table 9).

### 3.9. Evaluation of ODTs

#### 3.9.1. Content Uniformity

Content uniformity in the prepared batches of ODTs with microparticulate pellets was found to be between 98.36 ± 1.75–99.81 ± 1.01%, as shown in Table 10. Results indicate the uniform distribution of the drug in the tablets containing multiparticulates.

#### 3.9.2. Weight Variation

All the prepared ODT formulations showed the weight variation within limits as per the USP 39 requirements for weight variation, as shown in Table 10.

#### 3.9.3. Hardness and Friability

Hardness values of the ODT formulations were found in the range of 2.216 ± 0.654–2.538 ± 0.743 kg/cm^2^ (Table 10). The highest hardness value was observed for ODT 7b, with the mean value of 2.538 kg/cm^2^.

All the ODT formulations prepared with CL-PVP showed friability in the range of 0.712–1.296% (Table 10), and ODT 7b showed a friability of 0.712%.

Friability for ODTs prepared from CCS and SSG as disaggregating agents was found in the range of 0.692–1.268% and 0.680–1.270%, respectively (Table 10). The difference in the friability value may be due to differences in the excipient amount used in the formulation development.

#### 3.9.4. Wetting Time

According to the compendia standards, ODT should disintegrate within 3 min when examined via the test for the disintegration of tablets and capsules [37]. The average wetting time for the formula prepared with CL-PVP, i.e., ODT 5a, ODT 5b, ODT 6, ODT 7a, and ODT 7b, was found to be 12.05, 11.272, 12.08, 11.45, and 11.17 s, respectively, and showed better wetting time as compared to CCS and SSG (Table 10). Hence, CL-PVP can be a good superdisintegrating agent for ODT.

Furthermore, it was observed that the main factor influencing the wetting time of ODTs prepared with CL-PVP was the compression force rather than the disintegrant concentration, while, when SSG was used as a disintegrant, regardless of the compression force, the disintegration/wetting time was only dependent on the concentration of the disintegrant [38]. A similar observation was found with SSG containing ODT in this experiment. In addition, it was also noticed that during the wetting time experiment, ODTs containing SSG were swelled, and the outer edge appeared gel-like, whereas when the tablets contained CL-PVP, they hydrated quickly with faster de-aggregation as compared to ODTs prepared with CCS and SSG. Also, the center of the tablets with SSG and CCS remained dry and hard after complete wetting.

#### 3.9.5. In Vitro Dissolution Studies of ODTs

**Figure 9:**
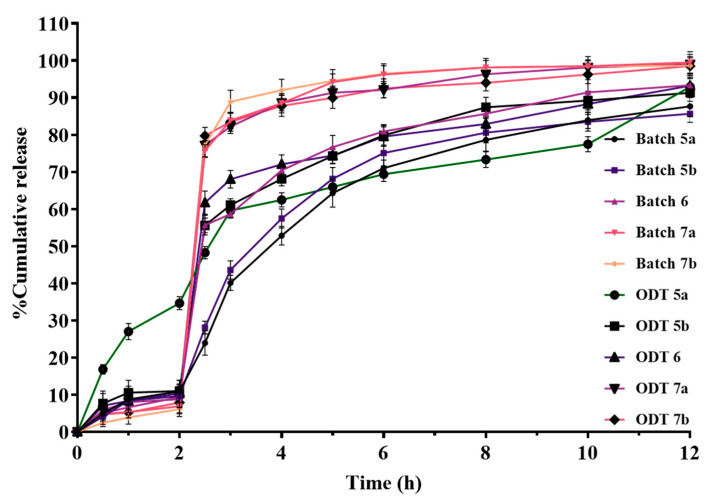
Comparative drug release from ODT prepared with selected multiparticulates pellet batches and super-disintegrating agent (CL-PVP).

The comparative release profiles of ODTs with selected multiparticulates pellets are shown in Figure 9. These pellets were mixed and compressed into the ODTs using direct compressible diluents and different superdisintegrants. Based on wetting time, ODTs prepared with CL-PVP superdisintegrant were selected for the in vitro release studies. Among all the prepared ODT formulations, ODT 5a showed the highest drug release (34.681%), while ODT 5b, ODT 6, ODT 7a, and ODT 7b showed 11.019%, 10.385%, 8.817%, and 7.904% drug release, respectively, in acidic medium for 2 h. Therefore, it can be concluded that of the selected multiparticulates batches, when compressed into ODT formulations, ODT 7a and ODT 7b exhibited the best in vitro release profile in an acidic medium or simulated gastric pH at 2 h. Also, ODT 7a and ODT 7b released more than 75% in phosphate buffer of pH 6.8 after 2.5 h.

#### 3.9.6. Kinetic Model for Drug Release Mechanism from ODT Formulations

Kinetic modeling of the release profile for various ODT formulations was performed to determine the underlying release mechanisms. The correlation coefficient values for various models are listed in Table 11. ODT 5a followed the Korsmeyer–Peppas release model, while ODT 5b, ODT 6, ODT 7a, and ODT 7b followed first-order release kinetics as per regression values (Table 11).

As expected from first-order release kinetics, the rate of drug release will progressively decrease over time, as the reservoir of drug in the device decreases [39]. Hence, the developed ODTs, except ODT 5a, showed the first-order release kinetics, as was confirmed by the release pattern as well as through the application of the release kinetics model.

## 4. Discussion

An enteric-coated multiparticulate system for the drug was developed by employing an inert core material using a bottom spray fluidized coater. Four steps were followed, for instance, a seal coat of ethyl cellulose on core material, drug layering on seal-coated core particles, and sub-coating on drug-layered core with different grades of hydrophilic polymer. Finally, an enteric coat was applied on sub-coated particles with pH-dependent polymer (Eudragit L100 or Eudragit L30 D-55). The pH-responsive polymers have ionizable acidic or basic residues whose ionization depends on the solution pH of the medium [40,41].

The enteric coat polymer resists the drug release in the stomach, i.e., prevents the release of the drug in the acidic medium, whereas it releases the drug into the intestine. Enteric-coated drug pellets follow a sustained release profile and improve clinical efficacy, patient compliance, and reduce the associated side effects. Furthermore, a multiparticulates system can deliver the incompatible agents simultaneously, homogeneous distribution throughout the GIT, augmented absorption of the drug, minimal local irritation, reduced risk of dose dumping, attainment of stable drug plasma levels, and reduce the inter- and intra-patient variability [42].

In this experiment, the enteric coating material is weakly acidic, while the pantoprazole sodium-loaded pellets are weakly basic; therefore, a sub-coating layer was added between them to improve the stability and acid resistance of the enteric-coated pellets. Hence, the pellets loaded with pantoprazole sodium were coated with hydroxypropyl methylcellulose (HPMC) as a separating layer.

In addition, the glass transition temperature of Eudragit^®^ L100-55 and Eudragit^®^ L30-D is approximately 110 °C; therefore, a plasticizer was added during the coating process to reduce the glass transition temperature of the material and adjust film toughness [43]. Also, the plasticizer in the coating material increases both the plasticity and permeability of the polymer material. In general, the amount of plasticizers with enteric coating polymer is approximately added 5 to 20% *w*/*w* during the coating process [7]. Thus, considering that the enteric coating film requires sufficient resistance to gastric acidic pH, the amount of plasticizer was selected 10% *w*/*w* of the total enteric coating material.

The particle size analysis showed a narrow size distribution, as it is evident from the low span values, and a single slender peak for the optimized batches of pellets (Table 4, Figure 1, Figure 2 and Figure 3). Also, the smaller span values stated for all pellet batches indicate their sphericity and lower size distribution deviation [44]. Results of the morphology analysis of the multiparticulates pellets are shown in Figure 4, Figure 5 and Figure 6. Roundness measures the sphericity, pellet elongation is defined by the aspect ratio, and rectang measures the rectangular shape of pellets, respectively, and for a perfect sphere, these shape parameters should have the value of unity [45]. The roundness factor for a circle is 1, the rectang (0.833), roundness (0.917), and elongation (1.135) of Batch 7b approach the value of unity (Table 5); hence, the developed pellets were formed almost circular (Figure 6b) [46].

The variation in density of pellets from batch to batch affects the potency of finished products, produces segregation during mixing, and causes problems in batch size determination during coating. The bulk and tapped densities of various batches of pellets, along with the Hausner ratios, Carr’s index, and porosity, are represented in Table 6. Bulk density indicates the extent of densification or compactness of a substance. Bulk density and porosity of the optimized Batch 7b were found 0.741 g/cm^3^ and 6.49%, respectively (Table 6). According to Trivedi, Knop, and colleagues, bulk densities of the prepared pellet batches should be high, while porosity should be low [47,48]. A high bulk density in drug pellets is advantageous as it enhances drug loading capacity within a specified volume, improves flowability, and results in more compact and stable dosage forms [49]. It is often ideal for drug-loaded pellets to have low porosity to control the drug release rate, raise the pellets’ mechanical strength, and increase their stability [50]. Due to the high porosity results in the faster drug release and dose dumping, and it may also make the pellets more brittle and prone to crushing [51,52].

The Hausner ratio and Carr’s index are measures of interparticle friction and have been widely used to estimate the flow properties. A Hausner ratio value of less than 1.20 indicates good flowability of the material, whereas a value of 1.5 or higher suggests poor flow of the material [53]. The optimized Batch 7b a showed Hausner ratio (1.017) and Carr’s index (6.443), as shown in Table 6; hence, the results exhibited excellent flow properties. Furthermore, the angle of repose (<30°) shows the excellent flow properties of the powder or granules (Table 7). All the batches exhibited good flow properties. The best flow properties were observed for optimized Batch 7b with an angle of repose of 18.29° as shown in Table 8.

The drug content was observed above 97% in all the developed multiparticulates (Table 8). Hence, the results indicated uniform loading of the drug in the pellets for all batches.

DSC analysis of the drug was performed to determine its thermal properties and the interactions with the excipients used in the multiparticulates formulations. The DSC results of optimized Batch 7b suggested that the drug-loaded multiparticulates pellets are composed of a homogeneous phase in which the polymer shows a higher degree of crystallinity than the drug. The results indicated that the drug may be molecularly dispersed in the sub-coating polymer (Figure 7).

Dissolution testing is intended to provide a step toward evaluating the physiological availability of the drug. For commercial products, dissolution testing is used primarily to confirm manufacturing and product consistency and to assess post-approval changes and the need for bioequivalence studies [54]. As per the pharmacopeia standard, the delayed-release formulations or enteric-coated formulations must not release the drug more than 10% in acidic media or 0.1 N HCl to ensure it reaches the intestines for release [55].

Figure 8 shows the dissolution profiles of all enteric-coated pellet batches. In acidic release media, it was observed that as enteric coating levels increased, the drug release in acidic medium (0.1 N HCl, pH 1.2) was observed at <10%. But at low percentage coating levels, the drug was released more than 10% in acidic medium over 2 h. Hence, the results indicate that sub-coating and enteric coating can play a crucial role in protecting the drug from degradation by hydrochloric acid in the stomach. Also, it was observed that with the high viscosity of HPMC E4M and HPMC K4M, the sub-coating process was time-consuming, because a large amount of solvent was required to reduce the viscosity for the smooth running of the process. Hence, we shifted to low viscosity sub-coating polymers (HPMC K100LV, HPMCE50, and HPMC E5) to reduce the processing time for sub-coating. Furthermore, batches (Batch 6, Batch 7a, and Batch 7b) prepared with these sub-coating polymers (Table 2) exhibited very low drug release in acidic medium, and nearly complete drug release was observed in phosphate buffer of pH 6.8 (Figure 8). In addition, Batch 7a and Batch 7b showed 6.877% and 6.136% of drug release in acidic medium at 2 h, respectively. The delayed release from these multiparticulates pellet batches in acidic medium might be due to optimal sub-coating and enteric coating levels. Moreover, high viscosity polymer sub-coating and non-aqueous enteric-coated multiparticulates batches demonstrated slower drug release (Figure 8). This may be because of the lower permeability of the dissolution medium into the non-aqueous enteric-coated multiparticulates pellets. Also, acrylic resin enteric coatings are denser and have lower permeabilities than cellulose enteric coatings in acidic environments [56]. The drug release in acidic medium was obtained in the following order: Batch 7b < Batch 7a < Batch 6 < Batch 5b < Batch 5a < Batch 4b < Batch 4a < Batch 3 < Batch 2 < Batch 1 (Figure 8). This could be due to the increase in successive sub-coating and enteric coating percentage levels. Hence, it can be concluded that the release of the drug was found to be directly proportional to the coating percentage of the sub-coating and enteric coating polymers.

In addition, kinetic modeling of the release profile for all batches was performed to determine the underlying release mechanisms. The correlation coefficient values for various models are listed in Table 9. All the batches exhibited non-Fickian release mechanisms because the value of the release exponent (n) was observed between 0.45 and 0.89 (Table 9) [57].

Out of all multiparticulates batches, Batch 5a, Batch 5b, Batch 6, Batch 7a, and Batch 7b confirmed the drug release in acidic medium at close to or less than 10% at 2 h (Figure 8), and found as per the pharmacopeia limits. Finally, these drug-loaded enteric-coated pellets were utilized further for the development of ODT and evaluated.

ODT should disintegrate rapidly in the mouth and possess enough structural integrity to withstand handling without substantial breakage. Thus, tablet properties such as hardness and friability are closely linked to causing rapid tablet disintegration. According to the European Pharmacopoeia IV (EP IV), all the formulations developed can be defined as ‘‘fast dispersible” if they de-aggregate within 3 min.

The drug content in the compressed ODTs was found to be >98%, which indicates the uniform distribution of the multiparticulates in the ODTs (Table 10). Hence, the results specify the uniform mixing of pellets with tablet blends and no segregation of pellets during the compression process. Weight variation was observed between 624.48 ± 2.134 to 628.52 ± 1.383 mg (Table 10), which was observed within the limit of 5% as per USP tablets’ weight variation limits (http://www.uspbpep.com/usp29/v29240/usp29nf24s0_c2091.html, accessed on 7 June 2025). ODT 7b showed the hardness of 2.538 kg/cm^2^, which was compressed with crosslinked PVP as a superdisintegrant, and found better than ODTs prepared with CCS and SSG (Table 10). It has been reported that the preferred strength of the orally disintegrating tablet is usually about 2–15 kg/cm^2^ [58]. For fast-disintegrating tablets, less hardness is required than conventional ones, due to a low compression force. Hence, these tablets may be fragile and need individual packaging.

In addition, tablet friability is a measurement of the tablet’s physical strength. As shown in Table 10, ODT 7b showed friability of 0.712%, which was lower than other ODT formulations. It was specified that an acceptable friability limit for dispersible tablets ranges from 0.95% to 1.5% [59,60]. Hence, ODT 7b showed friability within the limits (Table 10).

The wetting time of the tablets can be replaced with the disintegration time measurement. This experiment mimics the action of saliva in contact with the tablet [61,62]. To achieve quick disintegration, disaggregating (super-disintegrating) agents are added to the ODT formulations. In this experiment, CL-PVP, SSG, and CCS were utilized as superdisintegrants. These superdisintegrating agents act by the entry of liquid by wicking or capillary action which leads to swelling of the agent and pressure is exerted in the radial direction, therefore disintegrating the tablets [63]. The concentration of superdisintegrant agents employed in development of ODT formulations usually ranges between 10 and 30% *w*/*w*. The present ODT formulations contained 8.0–13.0% *w*/*w* of each superdisintegrant. ODT 7b, manufactured with 8% CL-PVP, showed a quicker wetting time/de-aggregation time (11.17 s) than an ODT formed with CCS (68.76 s) and SSG (73.58 s). This may be because the SSG works by a swelling mechanism, and the wetting time was observed to be longer. However, CL-PVP achieved its disintegrating action by wicking through capillary action, while CCS works by swelling and wicking actions. Therefore, it can be concluded that all ODTs prepared with CL-PVP showed faster wetting time than CCS and SSG (Table 10).

Hence, ODTs prepared with CL-PVP (Table 3) were tested for in vitro drug release studies, and the results are shown in Figure 9. According to all results of ODT formulations, drug release in acidic media of formulations ODT 7a and ODT 7b was observed less than 10% as compared to other ODT formulations (Figure 9). This could be due to the optimum level of sub-coating and aqueous enteric coating of the pellets providing a cushioning effect, and lead to a reduction in the crushing or cracking of the pellets during compression. Among all ODT formulations, ODT 7b showed the best result, as only 7.904% of the drug was released in the simulated gastric pH (0.1 N HCl) and about 79.749% in 2.5 h in phosphate buffer (pH 6.8). Both values were found well within the 10% drug release at 2 h in acidic medium and 75% or more drug release in 45 min in phosphate buffer, as per the USP Pharmacopoeia limits for delayed release formulations. Therefore, based on the hardness, friability, wetting time, and in vitro release studies, it can be concluded that the ODT 7b formulation containing Batch 7b pellets was found to be suitable among all prepared ODT formulations. In a nutshell, ODT 7b confirmed the hardness, friability, and wetting time of 2.234 kg/cm^2^, 0.712%, and 11.17 s, respectively. Further, ODT 7b showed the drug release in gastric pH and phosphate buffer at 2 h and 2.5 h, about 7.904% and 79.749%, respectively. Hence, it can be concluded that Batch 7b multiparticulates and ODT 7b formulation are the best formula with the current processing parameters for scaling-up purposes at an industrial scale.

## 5. Conclusions

We successfully developed enteric-coated multiparticulates containing pantoprazole sodium and formulated them into orally disintegrating tablets (ODTs). The physicochemical properties of both multiparticulates and ODTs were systematically evaluated. The multiparticulates, coated with HPMC E5 and Eudragit^®^ L30 D-55, demonstrated pH-dependent drug release—effectively minimizing release in acidic conditions and releasing the drug at intestinal pH, in compliance with pharmacopeial limits. The optimized ODT formulation (ODT 7b) exhibited favorable physicochemical characteristics and an in vitro release profile consistent with targeted release at both gastric pH (pH 1.2) and intestinal pH (pH 6.8). Among the tested disintegrants, the ODT containing Crospovidone M^®^ or CL-PVP showed the minimum wetting time while maintaining acceptable hardness and friability.

Therefore, it can be concluded that the ODT containing the developed multiparticulates represents a promising drug delivery system, offering several advantages including ease of administration, taste masking, sustained release, and enhanced bioavailability. The successful development and commercialization of this formulation requires careful consideration of formulation parameters, manufacturing processes, and quality control measures. Moreover, this type of dosage form may be particularly suitable for elderly and pediatric patients who have difficulty swallowing conventional solid oral formulations.

## Figures and Tables

**Table 2:** Different batch formulations with sub-coating and enteric coating materials onto drug-coated core pellets.

Formulation Code	Drug Layering (% *w*/*w*)	Sub-Coating Material	Sub- Coating (% *w*/*w*)	Enteric Coating Material	Enteric Coating (% *w*/*w*)
Batch 1	22.60	HPMC K4M	2.0	Eudragit L-100	10.0
Batch 2	22.60	HPMC E4M	3.0	Eudragit L-100	15.0
Batch 3	22.60	HPMC E4M	3.0	Eudragit L30 D-55	20.0
Batch 4a	22.60	HPMC E4M	4.0	Eudragit L30 D-55	25.0
Batch 4b	22.60	HPMC E4M	4.0	Eudragit L-100	35.0
Batch 5a	22.60	HPMC E4M	5.0	Eudragit L-100	35.0
Batch 5b	22.60	HPMC E4M	5.0	Eudragit L30 D-55	35.0
Batch 6	22.60	HPMC E50 and HPMC K100 LV (1:1)	6.0	Eudragit L30 D-55	35.0
Batch 7a	22.60	HPMC E5	10.0	Eudragit L30 D-55	35.0
Batch 7b	22.60	HPMC E5	15.0	Eudragit L30 D-55	35.0

**Table 3:** Compositions of ODT formulations.

Formulation Code	Drug * Containing Pellets (mg)	MTL DC (mg)	MCC PH102 (mg)	CL-PVP (mg)	CCS (mg)	SSG (mg)	Orange Flavor (mg)	Aspartame (mg)	Citric Acid (mg)	Mag. Stearate (mg)
ODT 5a	186.446	256.554	75.6	75.6	-	-	20.4	5.0	7.4	3.0
	186.446	256.554	75.6	-	75.6	-	20.4	5.0	7.4	3.0
	186.446	256.554	75.6	-	-	75.6	20.4	5.0	7.4	3.0
ODT 5b	160.0	264.1	94.5	75.6	-	-	20.4	5.0	7.4	3.0
	160.0	264.1	94.5	-	75.6	-	20.4	5.0	7.4	3.0
	160.0	264.1	94.5	-	-	75.6	20.4	5.0	7.4	3.0
ODT 6	199.576	224.524	94.5	75.6	-	-	20.4	5.0	7.4	3.0
	199.576	224.524	94.5	-	75.6	-	20.4	5.0	7.4	3.0
	199.576	224.524	94.5	-	-	75.6	20.4	5.0	7.4	3.0
ODT 7a	179.365	238.435	126.0	50.4	-	-	20.4	5.0	7.4	3.0
	179.365	238.435	126.0	-	50.4	-	20.4	5.0	7.4	3.0
	179.365	238.435	126.0	-	-	50.4	20.4	5.0	7.4	3.0
ODT 7b	186.884	230.916	126.0	50.4	-	-	20.4	5.0	7.4	3.0
	186.884	230.916	126.0	-	50.4	-	20.4	5.0	7.4	3.0
	186.884	230.916	126.0	-	-	50.4	20.4	5.0	7.4	3.0

* Pellets contained 22.6 mg of drug as sodium salt, which is equivalent to 20 mg of pantoprazole, mannitol DC (MTL DC), microcrystalline cellulose PH102 (MCC PH102), sodium starch glycollate (SSG, Explotab^®^), croscarmellose sodium (CCS, Ac-Di-Sol^®^), crosslinked polyvinylpyrrolidone (CL-PVP, Crosspovidon M^®^).

**Table 4:** Mean diameter and span values of various batches of multiparticulates.

Formulation Code	Mean Diameter (d 0.5, µm)	Span Value
Core material	174.631	0.640
Batch 1	197.671	0.646
Batch 2	356.300	0.834
Batch 3	439.299	0.735
Batch 4a	463.735	0.700
Batch 4b	519.184	0.645
Batch 5a	529.511	0.763
Batch 5b	525.395	0.653
Batch 6	452.488	0.634
Batch 7a	485.438	0.603
Batch 7b	509.139	0.625

**Table 5:** Shape factors of various batches of multiparticulates pellets.

Formulation Code	Elongation (Mean ± S.D.) *	Rectang (Mean ± S.D.) *	Roundness (Mean ± S.D.) *
Batch 1	1.253 ± 0.189	0.775 ± 0.016	0.798 ± 0.117
Batch 2	1.103 ± 0.048	0.790 ± 0.034	0.814 ± 0.030
Batch 3	1.098 ± 0.043	0.793 ± 0.006	0.792 ± 0.022
Batch 4a	1.197 ± 0.058	0.830 ± 0.036	0.903 ± 0.043
Batch 4b	1.298 ± 0.062	0.768 ± 0.050	0.781 ± 0.049
Batch 5a	1.239 ± 0.021	0.865 ± 0.119	0.801 ± 0.064
Batch 5b	1.112 ± 0.030	0.779 ± 0.028	0.885 ± 0.053
Batch 6	1.162 ± 0.122	0.766 ± 0.008	0.852 ± 0.057
Batch 7a	1.12 ± 0.079	0.799 ± 0.054	0.903 ± 0.014
Batch 7b	1.135 ± 0.051	0.833 ± 0.043	0.917 ± 0.053

* n = 3, S.D.—standard deviation.

**Table 6:** Bulk density, tapped density, Hausner ratio, Carr’s index, and porosity data.

Formulation Code	Bulk Density (g/cm^3^)	Tapped Density (g/cm^3^)	Hausner’s Ratio	Carr’s Index	Porosity (%)
Batch 1	0.689 ± 0.012	0.783 ± 0.01	1.136	12.382	9.57
Batch 2	0.680 ± 0.008	0.776 ± 0.011	1.127	11.635	9.96
Batch 3	0.677 ± 0.014	0.741 ± 0.015	1.113	9.481	10.14
Batch 4a	0.704 ± 0.01	0.769 ± 0.012	1.092	8.474	8.45
Batch 4b	0.684 ± 0.01	0.763± 0.007	1.142	12.257	9.21
Batch 5a	0.733 ± 0.009	0.805 ± 0.012	1.141	13.362	10.52
Batch 5b	0.798 ± 0.01	0.869 ± 0.008	1.090	8.286	8.24
Batch 6	0.782 ± 0.003	0.835 ± 0.003	1.059	5.582	5.57
Batch 7a	0.745 ± 0.01	0.808 ± 0.005	1.085	7.791	7.75
Batch 7b	0.741 ± 0.004	0.792 ± 0.008	1.017	6.443	6.49

**Table 7:** Standard limits of Carr’s index, Hausner ratio, and angle of repose for flow properties.

Carr’s Index	Hausner Ratio	Angle of Repose	Flow Properties
≤10	1.0–1.11	25–30	Excellent
11–15	1.12–1.18	31–35	Good
16–20	1.19–1.25	36–40	Fair
21–25	1.26–1.34	41–45	Passable
26–31	1.35–1.45	46–55	Poor
32–37	1.46–1.59	56–65	Very poor
>38	>1.60	>66	Very very poor

**Table 8:** Angle of repose and flow rate of various batches of pellets.

Formulation Code	Angle of Repose (Degree)	Flow Rate (g/s)	Drug Content (%) ± SD
Batch 1	24.78 ± 0.24	2.63 ± 0.025	97.83 ± 1.41
Batch 2	25.08 ± 0.23	2.32 ± 0.033	98.36 ± 1.23
Batch 3	21.34 ± 0.46	3.55 ± 0.045	99.02 ± 1.15
Batch 4a	22.01 ± 0.19	3.30 ± 0.023	99.78 ± 1.28
Batch 4b	26.12 ± 0.32	2.43 ± 0.026	98.64 ± 1.21
Batch 5a	27.85 ± 0.24	2.07 ± 0.019	98.33 ± 1.31
Batch 5b	22.82 ± 0.39	3.69 ± 0.034	99.47 ± 1.17
Batch 6	21.68 ± 0.21	3.86 ± 0.024	98.22 ± 1.24
Batch 7a	19.85 ± 0.17	4.05 ± 0.031	99.24 ± 1.14
Batch 7b	18.29 ± 0.34	4.16 ± 0.029	99.51 ± 1.15

**Table 9:** An overview of the different kinetic release models followed by various batches of multiparticulates pellets.

Formulation Code	r^2^ Values						Best Fit Model
	Zero- Order (ZO)	First- Order (FO)	Hixson-Crowell (HC)	Korsmeyer–Peppas (KP)		Higuchi	
				r^2^	Slope (n)		
Batch 1	0.503	0.619	0.461	0.812	0.649	0.674	KP
Batch 2	0.556	0.723	0.499	0.832	0.774	0.719	KP
Batch 3	0.846	0.801	0.568	0.871	0.858	0.797	KP
Batch 4a	0.752	0.887	0.657	0.918	0.883	0.876	
Batch 4b	0.763	0.889	0.682	0.888	0.950	0.864	FO
Batch 5a	0.833	0.930	0.734	0.927	0.992	0.917	FO
Batch 5b	0.825	0.944	0.716	0.907	1.058	0.910	FO
Batch 6	0.753	0.954	0.637	0.849	1.072	0.863	FO
Batch 7a	0.718	0.959	0.602	0.805	1.141	0.833	FO
Batch 7b	0.687	0.954	0.577	0.819	1.337	0.812	FO

**Table 10:** Evaluation parameters of ODTs.

Formulation Code	Diameter and Thickness (mm)	Average Weight ± S.D. (mg)	Hardness ± S.D. (kg/cm^2^)	Friability (%)	Wetting Time ± S.D. (s) n = 3	% of Drug Content
ODT 5a	13.219 ± 0.042, 4.677 ± 0.035	624.48 ± 2.134	2.418 ± 0.164	1.064	12.05 ± 1.061	98.36 ± 1.75
	13.214 ± 0.066, 4.688 ± 0.054	626.39 ± 2.242	2.385 ± 0.443	1.242	62.23 ± 1.842	98.92 ± 1.43
	13.192 ± 0.075, 4.674 ± 0.035	624.64 ± 2.445	2.216 ± 0.654	1.236	69.61 ± 1.754	99.18 ± 2.19
ODT 5b	13.217 ± 0.047, 4.682 ± 0.045	626.78 ± 2.442	2.343 ± 0.271	1.163	11.272 ± 1.822	99.13 ± 1.77
	13.175 ± 0.045, 4.689 ± 0.042	625.57 ± 2.863	2.472 ± 0.476	1.201	58.65 ± 1.098	99.08 ± 1.54
	13.190 ± 0.049, 4.693 ± 0.041	626.86 ± 2.688	2.428 ± 0.689	1.214	66.84 ± 1.688	99.31 ± 1.72
ODT 6	13.184 ± 0.056, 4.645 ± 0.054	627.27 ± 2.935	2.465 ± 0.344	0.801	12.08 ± 1.038	98.72 ± 1.33
	13.177 ± 0.048, 4.642 ± 0.065	625.78 ± 2.684	2.508 ± 0.743	0.758	56.82 ± 1.841	99.18 ± 1.52
	13.211 ± 0.049, 4.619 ± 0.073	626.67 ± 2.363	2.497 ± 0.545	0.764	60.63 ± 1.692	99.25 ± 1.26
ODT 7a	13.208 ± 0.063, 4.634 ± 0.062	627.92 ± 2.524	2.524 ± 0.256	0.718	11.45 ± 1.028	99.03 ± 1.64
	13.203 ± 0.068, 4.628 ± 0.083	626.86 ± 2.282	2.452 ± 0.562	0.733	69.43 ± 1.741	98.66 ± 1.53
	13.212 ± 0.067, 4.629 ± 0.075	624.98 ± 2.644	2.483 ± 0.618	0.729	74.76 ± 1.583	98.52 ± 1.30
ODT 7b	13.157 ± 0.075, 4.611 ± 0.056	628.24 ± 1.864	2.538 ± 0.144	0.712	11.17 ± 1.051	99.81 ± 1.01
	13.162 ± 0.035, 4.609 ± 0.068	627.44 ± 2.083	2.376 ± 0.446	0.692	68.76 ± 1.558	99.13 ± 1.68
	13.159 ± 0.066, 4.617 ± 0.054	628.52 ± 1.383	2.298 ± 0.554	0.680	73.58 ± 1.629	99.21 ± 1.35

**Table 11:** An overview of the different kinetic models, followed by ODT formulations with selected multiparticulate pellet batches.

Formulation Code	r^2^ Values						Best Fit Model
	Zero-Order (ZO)	First-Order (FO)	Hixson-Crowell (HC)	Korsmeyer–Peppas (KP)		Higuchi	
				r^2^	Slope (n)		
ODT 5a	0.801	0.934	0.706	0.945	0.501	0.919	KP
ODT 5b	0.790	0.933	0.687	0.885	0.878	0.891	FO
ODT 6	0.772	0.959	0.656	0.846	0.960	0.875	FO
ODT 7a	0.724	0.944	0.615	0.832	1.013	0.840	FO
ODT 7b	0.695	0.947	0.589	0.818	1.137	0.818	FO

## Data Availability

The data supporting the findings of this study are available from the corresponding author upon reasonable request.
